# Diagnostic role of medical thoracoscopy in childhood pleural tuberculosis

**DOI:** 10.1038/s41598-019-44860-6

**Published:** 2019-06-10

**Authors:** Maoshui Wang, Chao Han, Yu He

**Affiliations:** 1 Department of Pediatrics, Qilu Hospital, Shandong University, Jinan, Shandong China; 2 Department of Lab Medicine, Shandong Provincial Chest Hospital, Shandong University, Jinan, Shandong China; 3 Department of Geriatrics, Shandong Mental Health Center, Jinan, China; 4 Department of Clinical Laboratory, First Affiliated Hospital of Guangxi Medical University, Nanning, Guangxi China

**Keywords:** Paediatric research, Paediatric research

## Abstract

Currently, the diagnostic role of medical thoracoscopy in childhood pleural tuberculosis remains uncertain. Therefore, this retrospective study was conducted to evaluate the diagnostic performance of histological examination of tissue samples obtained by medical thoracoscopy in childhood pleural tuberculosis. Hospitalized children who underwent medical thoracoscopy between May 2012 and March 2016 were included in the study. Tissue samples obtained by thoracoscopy were submitted for histological examination (hematoxylin and eosin staining). Descriptive statistical methods were used for data interpretation, and the data were expressed as the mean ± standard deviation. The childhood tuberculosis patients had the following characteristics: 11 had pleural tuberculosis and 9 had pleural tuberculosis + pulmonary tuberculosis, the average age was 13.0 ± 2.2 years old, 60% were male, 26.3% (5/19) of patients tested positive for acid-fast bacilli positive in smears, 21.1% (4/19) of patients were positive for TB-PCR, and 90% (18) of pleural tuberculosis patients were positive in the culture for *Mycobacterium tuberculosis*. The sensitivity of histological examinations of tissue samples obtained by thoracoscopy in the detection of pleural tuberculosis was 80% (16/20). Complications were reported in 15 cases, and all complications disappeared spontaneously without any specific treatment. Therefore, we concluded that medical thoracoscopy was a sensitive and safe tool for the detection of childhood pleural tuberculosis.

## Introduction

Tuberculosis (TB) remains one of the world’s most serious infectious diseases. Approximately one-third of the human population is infected with *Mycobacterium tuberculosis* (*M*.*TB*), with 9.6 million new cases and 1.7 million deaths per year^1^. China ranks second among the 22 high-burden countries for TB. In 2014, the WHO reported that 1.3 million new cases of TB were estimated to occur. For childhood TB, it was estimated that 7.6 million children were infected by *M*.*TB*, and one-tenth developed the disease^2^. Despite causing morbidity and mortality, childhood TB remains a neglected disease: (1) childhood TB, which is paucibacillary and smear-negative, is not prioritized by national TB control programs; (2) current TB assays have performed poorly in the detection of childhood TB; and (3) obtaining specimens for TB examination is difficult. Therefore, more research, prevention and diagnosis of TB among children are urgently needed.

In a previous study, we described the clinicopathologic characteristics of childhood pleural TB patients^3^. The data showed that TB assays, such as acid-fast bacilli (AFB) smears, PCR and mycobacterial cultures, have limited diagnostic value in childhood pleural TB. Timely diagnosis is critical to ensure optimized therapy and avoid unnecessary treatment. The ideal methods of diagnosing TB are histological examination and mycobacterial culture. However, often in children this is not possible due to the low bacterial load and difficulty in collecting specimens^4–6^.

Medical thoracoscopy has proved to be an effective and safe procedure for diagnosing pleural effusions of undetermined causes and is particularly helpful in the detection of tuberculous pleural effusion in high TB prevalence areas^7^. However, the diagnostic role of medical thoracoscopy in childhood pleural tuberculosis remains uncertain. In the study, our goal was to evaluate the diagnostic performance of histological examination of tissue samples obtained by thoracoscopy in the detection of childhood pleural TB.

## Patients and Methods

The study was conducted at the Department of Laboratory Medicine, Shandong Provincial Chest Hospital (SPCH). Ethical approval was provided by the ethics committee of SPCH. All patients gave informed consent to undergo thoracoscopy. However, informed consent was waived for the study by the ethics committee of SPCH given the retrospective study design. All patient records were anonymized and de-identified prior to analysis. The SPCH is one of the largest provincial referral TB hospitals in China.

Between May 2012 and March 2016, hospitalized children who underwent medical thoracoscopy were included in the study. Defined pleural TB cases were diagnosed if mycobacterial culture or TB-PCR was positive or if histological examination was positive. Probable cases were diagnosed based on AFB smears, and a response to anti-TB medications was revealed by clinical symptoms and chest radiographs. A control subject was defined if an alternative diagnosis was made.

Briefly, thoracoscopy was performed in the lateral decubitus position. Preoperative thoracic ultrasounds (Logiq 5, GE, Waskesha, USA) were performed to evaluate pleural adhesions, thickenings, nodules and masses and were also suggested to identify the optimal site of entry. After local or total anesthesia, an incision of approximately 1 cm was made, and a 5-mm thoracoscope (Richard Wolf, Knittlingen, Germany) was inserted via a 5-mm trocar. The pleural tissues were retrieved with grasping forceps. Abnormal areas, such as those exhibiting hyperemia, edema and a diffuse, single nodule, were biopsied. Usually, 3 or 5 biopsy specimens with 1 cm to 2 cm in margins were obtained. The chest tubes were left routinely.

Tissues were submitted for histological examination (hematoxylin and eosin staining), and each case was reviewed independently by two pathologists. The histopathological examination of pleural biopsies was reported as probable or definite TB according to the findings of epithelioid cells, multinucleated giant cells or caseous necrosis (definite). Mycobacterial cultures (Löwenstein-Jensen method), AFB (Auramine O stain) and TB-PCR (DAAN, Guangzhou, China) were conducted on several types of specimens, such as sputum, pleural effusions and tissues. It was worth noting that fewer than 2% of cultures became contaminated in the laboratory, and until now, none of tissue cultures contaminated. Pleural biochemistry tests, such as total protein (Siemens, Tarrytown, NY, USA), total bilirubin (Siemens, Tarrytown, NY, USA), glucose (Siemens, Tarrytown, NY, USA), lactate dehydrogenase (Kehua, Shanghai, China) and adenosine deaminase (Maker, Chengdou, Sichuan, China), were assayed on an Advia 2400 chemistry analyzer (Siemens, Tarrytown, NY, USA). Data were expressed as the mean ± standard deviation (SD), and the sensitivities of the histological examination of tissue samples obtained by medical thoracoscopy in the detection of pleural TB were calculated. The data that support the findings of this study are available from the corresponding author on reasonable request.

## Results

### Patient characteristics

**Figure 1:**
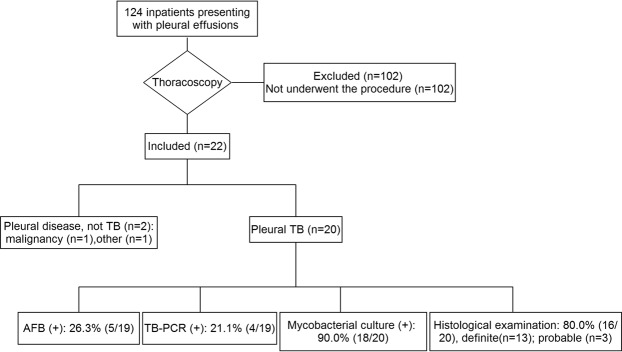
Flow diagram of the selected population.

As shown in Table 1, a total 22 patients were enrolled in our study (Fig. 1). Among them, two patients were diagnosed as pleural disease, not TB (one was lung cancer and the other was interstitial lung disease), and the remaining 20 patients were pleural TB, including 11 with pleural TB and 9 with pleural TB + pulmonary TB. Thirteen (59.1%, 13/22) patients were tested for HIV, and all were seronegative. For the 20 pleural TB patients, the average age was 13.0 ± 2.2 years; 60% (12/20) were male; and fever was the most common presenting symptom (90%, 18/20), followed by chest pain (55%, 11/20), cough (45%, 9/20) and dyspnea (20%, 4/20). Two patients had a contact history with TB patients. The individual patient characteristics were included in Table 2.

**Table 1:** Summary of characteristics of enrolled children patients.

	Total (n)	Pleural TB (n)
Characteristics		
Number	22	20
Sex, male	59.1% (13/22)	12/20 (60%)
Age (years)	13.0 ± 2.2	13.0 ± 2.2
HIV (−)	100% (13/13)	
TB contact history	2	2
Comorbidities		
Pleural TB alone	9	9
Pleural + pulmonary TB	11	11
Symptoms		
fever	82% (18/22)	90% (18/20)
chest pain	55% (12/22)	55% (11/20)
cough	45% (10/22)	45% (9/20)
dyspnea	23% (5/22)	20% (4/20)
Pleural analysis		
Total protein (g/L)	48.9 ± 5.6	
Total bilirubin (mmol/L)	11.27 ± 10.56	
Glucose (mmol/L)	3.17 ± 1.57	
Lactate dehydrogenase (U/L)	1021 ± 843	
Adenosine deaminase (U/L)	62.5 ± 32.4	
Thoracoscopic findings		
Loculation	72.7% (16/22),	
Adhesion	72.7% (16/22)	
Pleural thickening	86.4% (19/22)	
Diagnostic performance for TB		
AFB smear		26.3% (5/19)
TB-PCR		21.1% (4/19)
Mycobacterial culture		90% (18/20)
Pathological evidence for TB		80% (16/20)
Complications		
Significant pain	54.5% (12/22)	
Bleeding	27.3% (6/22)	
Nausea	4.5% (1/22)	
Pneumothorax	4.5% (1/22)

**Table 2:** Results of histological examination and demographic, clinical characteristics of all patients.

Cases	Sex	Age (years)	Symptoms	Time before admission	Contact history	Effusion site	HIV	AFB	Mycobacterial culture	TB-PCR	Histological examination	Diseases
Case 1	M	7	Fever	20 days	Yes	Left	N	SP (−)	SP (−), T (+)	T (−)	Probable TB	Pleural + Pulmonary TB
Case 2	F	8	Fever, Chest pain	1 month	Yes	Right	—	—	PE (+), T (+)	PE (−), T (−)	Definite TB	Pleural TB
Case 3	F	11	Fever	40 days	No	Left	N	SP (−), PE (−), T (−)	SP (−), P (+), T (+)	—	Definite TB	Pleural TB
Case 4	M	12	Fever, Cough, Chest pain	7 days	No	Right	N	SP (−), PE (−), T (+)	SP (−), PE (−), T (+)	PE (−)	Definite TB	Pleural + Pulmonary TB
Case 5	M	12	Fever, Chest pain	7 days	No	Right	N	SP (−), PE (−), T (+)	SP (+), PE (+), T (+)	PE (−)	Definite TB	Pleural TB
Case 6	M	12	Fever, Cough, Dyspnea, Chest pain	13 days	No	Right	—	T (−)	T (−)	T (−)	N	Pleural TB
Case 7	M	13	Fever	10 days	No	Right	N	PE (−), T (−)	PE (−), T (+)	PE (−), T (−)	Probable TB	Pleural TB
Case 8	F	13	Fever, Cough	1 month	No	Right	—	SP (−), T (+)	SP (+), T (+)	SP (−), T (+)	Definite TB	Pleural + Pulmonary TB
Case 9	F	13	Dyspnea, Chest pain	1 month	No	Left	N	SP (−), PE (−), T (−)	SP (−), PE (−), T (+)	SP (−)	Definite TB	Pleural TB
Case 10	M	13	Fever, Cough, Dyspnea, Chest pain	15 days	No	Right	N	SP (−), PE (−), T (−)	SP (−), PE (+), T (+)	SP (−), PE (+), T (−)	Definite TB	Pleural TB
Case 11	M	14	Fever, Cough, Chest pain	1 month	No	Left	N	SP (−), T (−)	SP (+), T (−)	SP (−), T (−)	Probable TB	Pleural + Pulmonary TB
Case 12	F	14	Fever, Chest pain	1 month	No	Left	N	SP (−), PE (−), T (−)	SP (−), PE (−), T (+)	P (−), T (−)	Definite TB	Pleural + Pulmonary TB
Case 13	F	14	Fever, Cough	1 month	No	Both	—	SP (−), T (+)	SP (+), T (+)	SP (−), T (+)	Definite TB	Pleural + Pulmonary TB
Case 14	M	14	Chest pain	1 year	No	Right	N	SP (−), PE (−), T (+)	SP (+), PE (+), T (+)	P (−)	Definite TB	Pleural TB
Case 15	F	14	Fever, Cough	10 days	No	Right	N	SP (−)	T (+)	T (+)	N	Pleural TB
Case 16	F	15	Fever	10 days	No	Right	—	PE (−), T (−)	T (−)	PE (−)	N	Pleural TB
Case 17	M	15	Fever, Chest pain	1 month	No	Right	—	SP (−), T (−)	SP (−), T (+)	PE (−), T (−)	Definite TB	Pleural + Pulmonary TB
Case 18	M	15	Fever, Cough	20 days	No	Both	N	SP (−)	SP (−), T (+)	SP (−)	Definite TB	Pleural TB
Case 19	M	15	Fever, Chest pain	5 days	No	Left	N	SP (−)	SP (+), T (+)	SP (−)	Definite TB	Pleural + Pulmonary TB
Case 20	M	15	Fever, Cough, Dyspnea	1 month	No	Both	—	SP (−), T (−)	SP (−), PE (−), T (+)	SP (−), PE (−), T (−)	N	Pleural + Pulmonary TB
Case 21	F	12	Cough, Dyspnea	13 days	No	Left	N	SP (−), PE (−), T (−)	SP (−), PE (−), T (−)	PE (−), T (−)	N	Lung cancer, MPE
Case 22	M	15	Dyspnea, Chest pain	6 days	No	—	N	SP (−), T (−)	SP (−), T (−)	T (−)	N	Pneumothorax, Pneumatocele, Interstitial Lung Disease

HIV, human immunodeficiency virus; AFB, acid-fast bacilli; SP, sputum; PE, pleural effusion; T, tissue; TB-PCR, tuberculosis-polymerase chain reaction; M, male; F, female; N, negative; TB tuberculosis; MPE, malignant pleural effusion.

### Pleural analysis

Pleural effusions from 14 patients (including one pleural disease, not TB) were evaluated for biochemistry and all were classified as exudates. Pleural cytology was evaluated in 7 patients (including one pleural disease, not TB) and all were lymphocyte-dominant. Biochemical analysis was performed on pleural effusions in pleural TB, total protein was 48.9 ± 5.6 g/L, and total bilirubin was 11.27 ± 10.56 mmol/L. Glucose was 3.17 ± 1.57 mmol/L, lactate dehydrogenase was 1021 ± 843 U/L, and adenosine deaminase was 62.5 ± 32.4 U/L.

### Thoracoscopic findings

Fifteen patients were locally anesthetized with 1% lidocaine, and the remaining seven were anesthetized with intravenous fentanyl citrate (0.1 mg/kg intravenously). Seventeen patients have detailed clinical information on the procedure: two patients were biopsied from visceral pleura, 15 from parietal pleura and the locations of the biopsied tissues from the remaining five were unclear. Thoracoscopic findings showed loculation in 16 cases (72.7%, 16/22), adhesion in 16 cases (72.7%, 16/22) and pleural thickening in 19 cases (86.4%, 19/22).

### Diagnostic performance of medical thoracoscopy

In the study, sensitivity was calculated using the clinical diagnosis as the reference: 26.3% (5/19) of pleural TB patients were classified AFB positive from their smears, TB-PCR yielded positive results in 21.1% (4/19) of pleural TB patients, and 90% of pleural TB patients (18/20) were positive in culture for *M*.*TB*. The sensitivity of histological examinations of tissue samples obtained by medical thoracoscopy in the detection of pleural TB was 80% (16/20), including 13 definite cases and 3 probable cases.

### Complications and outcomes

Complications of thoracoscopy were reported in 15 cases, including postoperative pain (12 cases, 54.5%), mild bleeding (6 cases, 27.3%), nausea (1 case, 4.5%) and pneumothorax (1 case, 4.5%). All complications disappeared spontaneously without any specific treatment. None of the TB patients required thoracic surgery or developed infections.

Pleural tissues were taken from all patients, and nine children (9/22, 40.9%) subsequently underwent an interventional procedure (such as decortication of pleural fibrous tissue and peeling off the additional adhesions) using thoracoscopy. Of the nine children, four (cases 5, 8, 13 and 14) had a repeat thoracoscopy for intervention. Although anti-TB treatment had been completed in case 9, a relapse occurred. Case 22 underwent thoracoscopic surgery for pulmonary bulla.

## Discussion

After several decades of neglect, childhood TB is receiving some of the attention it deserves. In 2014, the WHO developed the Roadmap for Childhood TB. Its plan, to reach zero TB deaths among children worldwide is within our grasp^8^. There are several reasons childhood TB has been neglected. Most important is the misconception that childhood TB is difficult to diagnose because it can be culture-confirmed in only 20% to 40% of cases (compared with 90% of adult cases)^9^. As a result, many childhood TB cases are never diagnosed and most children are diagnosed clinically. This would cause a significant delay in the diagnosis of childhood TB. In addition, diagnostic accuracy has a significant impact on the successful treatment of childhood TB.

Histology can provide strong supportive evidence for a diagnosis of TB. Histological examination has several advantages, such timeline, low cost, and no need for special equipment. However, the accuracy of histological examination is dependent on obtaining an adequate tissue sample^10^. Several tissue sampling techniques have been evaluated in children for TB diagnosis, such as, endobronchial ultrasound-guided transbronchial needle aspiration^11^, ultrasound-guided biopsy^12,13^, and CT-guided biopsy^14^.

For pleural disease, thoracoscopy seems to be a safe, simple, and accurate tool^15,16^. In tuberculous pleuritis, the combined sensitivity of histology and culture for rigid thoracoscopy was nearly 100%^17,18^. In our study, we showed the technique has a sensitivity of 80%. It was a sensitive diagnostic method in detecting childhood pleural TB in high TB burden country. Similarly, a small sample size was used to conduct research in children in a low-TB burden country^19^. That study reviewed the results of seven children examined by medical thoracoscopy, and proposed a similar conclusion as that in this study. Although several types of complications have been reported in our study, only one child required further therapy for pneumothorax.

It should be worthy to be noted that CT- or ultrasound-guided biopsy have been demonstrated to be useful for determining the causes of pleural effusions^20–22^, and it has been suggested that the image-guided pleural needle biopsy has advantages in some aspects^22,23^. However, there were no randomized research studies performed in children, and the technique may not be applicable to childhood pleural TB.

Several limits should be taken into account when interpreting these results. First, this study has a retrospective nature and incomplete information about the patient’s characteristics. Second, the sample size was small, and few patients in the control group underwent thoracoscopy, because our hospital is a referral TB hospital. Third, unstandardised thoracoscopy was performed in the study, and this could lead to a biased sample set and uncertain generalisability of the results. Fourth, for detection of childhood pleural TB, further analysis must be performed to validate the role of CT in assessing childhood TB prior to thoracoscopy and to compare diagnostic values of thoracoscopy and image-guided pleural biopsy^24^.

## Conclusions

In conclusion, in a high-TB burden country, medical thoracoscopy was a sensitive and safe tool in the detection of childhood pleural TB.
